# Targeting N-cadherin (CDH2) and the malignant bone marrow microenvironment in acute leukaemia

**DOI:** 10.1017/erm.2023.13

**Published:** 2023-05-03

**Authors:** Jessica Parker, Sean Hockney, Orest W. Blaschuk, Deepali Pal

**Affiliations:** 1 Department of Applied Sciences, Northumbria University, Newcastle upon Tyne NE1 8ST, UK; 2 Zonula Incorporated, Kirkland, QC H9J 2X2, Canada; 3 Wolfson Childhood Cancer Research Centre, Translational and Clinical Research Institute, Faculty of Medical Sciences, Newcastle University, Herschel Building Level 6, Brewery Lane, Newcastle upon Tyne NE1 7RU, UK

**Keywords:** Anti-cancer drugs, cancer microenvironment, CDH2, leukaemia, treatment resistance

## Abstract

This review discusses current research on acute paediatric leukaemia, the leukaemic bone marrow (BM) microenvironment and recently discovered therapeutic opportunities to target leukaemia–niche interactions. The tumour microenvironment plays an integral role in conferring treatment resistance to leukaemia cells, this poses as a key clinical challenge that hinders management of this disease. Here we focus on the role of the cell adhesion molecule N-cadherin (CDH2) within the malignant BM microenvironment and associated signalling pathways that may bear promise as therapeutic targets. Additionally, we discuss microenvironment-driven treatment resistance and relapse, and elaborate the role of CDH2-mediated cancer cell protection from chemotherapy. Finally, we review emerging therapeutic approaches that directly target CDH2-mediated adhesive interactions between the BM cells and leukaemia cells.

## Background

Leukaemia accounts for 31% of cancer diagnoses in children up to 14 years of age in the UK, of which 401 children are diagnosed with acute lymphoblastic leukaemia (ALL) and 79 with acute myeloid leukaemia (AML) per year (Ref. 1); overall, the 5-year survival of ALL and AML is over 90 and 67%, respectively (Refs 1, 2). N-cadherin (CDH2) is a cell adhesion molecule that mediates adhesive interactions between leukaemia cells and the cells of the bone marrow (BM) (Refs 3, 4). These interactions facilitate leukaemia cell survival, evasion from apoptosis and cell dormancy ultimately resulting in treatment resistance (Refs 3, 4). Indeed, the niche-protected, dormant, non-apoptotic leukaemia cells may re-emerge in relapsed cases to develop resistance to therapy (Refs 5, 6).

Dysregulation of normal blood homoeostasis is the main underlying developmental anomaly that leads to ALL and AML. Leukaemogenesis usually comprises a series of steps with an accumulation of genetic and epigenetic changes, inducing extensive alterations impacting cell growth, metabolism, cell cycle progression, cell death and differentiation, leading to preleukaemic haematopoietic stem cells (HSCs) and subsequently the development of ALL and AML (Refs 7, 8, 9). Because of the complexity of epigenetic and genetic mutations, it is not fully understood what cascade of events occur to give rise to the leukaemia phenotype and which perturbations are responsible for driving leukaemogenesis. Through advancement of technology, single-cell RNA sequencing has become more accurate, and been applied to examine leukaemia cells at the transcriptional level. A study by Watcham *et al*. (Ref. 10) presented data that suggest many leukaemia perturbations can gain advantage over wild-type cells, and drive cells into a more active state (Ref. 10). Indeed, many studies show that in utero mutations are becoming more recognised as commonplace in acute leukaemia and could be responsible for fusion genes in paediatric patients with ALL and AML; these mutations are known as an initiating event (Refs 11, 12, 13). Fusion genes are chromosomal aberrations that have a role in leukaemogenesis (Ref. 14), and can involve genes associated with protein kinase pathways, transcription factor and epigenetic modifications (Ref. 11).

Across mammals the number of HSCs per individual is thought to be conserved, with approximately 300 HSCs at birth, compared with between 11 000 and 22 000 in adults. Development of childhood leukaemia depends on initial somatic mutations in HSCs, and because of the small HSC pool size these mutations are more likely to have a greater impact on the HSC population (Ref. 15). The Knudson ‘two-hit’ hypothesis, established in 1971, suggested that dominantly inherited predisposition to cancer begins with a germline mutation, as can be seen with fusion genes; however a second, somatic mutation is needed for tumourigenesis (Ref. 16).

For example, only about 1% of children born with the ETV6-RUNX1 fusion gene develop the second-hit mutation that is needed to transform to ALL, indicating the fusion gene mutation is weakly penetrant (Ref. 17). Many somatic mutations such as *TP53*, *RUNX1* and *IKZF1* are found at the same sites of germline mutations in children who develop leukaemia (Refs 18, 19, 20). For example, a germline mutation at *CEBPA* leads to the development of AML with almost complete penetrance, this mutation is known to present favourable outcome (Ref. 21).

Intensification of chemotherapeutic regimens is thought to be one of the main reasons for increased survival in childhood leukaemia; however, such treatment is associated with high morbidity and mortality rates. For example, in AML, high dose cytarabines used in young adults (15–24 years old) were reported to have a benefit to outcome; however, these results could not be translated to paediatric patients. The COG trial AAML1031 intensified induction chemotherapy with mitoxantrone and cytarabine and found that intensification did not achieve a survival benefit in paediatric patients, since remission rates were comparable with the AAML0531 trail which did not include intensifying induction chemotherapy. Moreover, additional haematological toxicity was found to be associated with treatment intensification, therefore showing an increased toxicity without any proportional benefit in treatment (Ref. 22).

Studies have been conducted worldwide to analyse toxicity of paediatric acute leukaemia treatment (Refs 23, 24, 25). Table 1 shows comparisons of different paediatric ALL protocols in the UK and two European countries and their associated toxicities. Results show that up to 49% of patients experienced an adverse event because of the chemotherapeutic agents used in their treatment, with toxicity-induced mortality rates up to 3.7%. The children studied by Zawitkowska *et al*. (Ref. 24) were evaluated for the ‘grade’ of toxicity; it was found that children with grade 3 or higher were found to have a lower overall survival and event-free survival rate compared with children with a lower grade of treatment toxicity (Ref. 24).

**Table 1.** Comparison of paediatric ALL protocols from the UK and two European countries, including study size and number of patients affected by adverse events

Study	Hough *et al*. (Ref. 23)	Zawitkowska *et al*. (Ref. 24)	Franca *et al*. (Ref. 25)
Study location	UK	Poland	Italy
Study size (patient numbers)	3126	1872	508
Total adverse events (AEs)	1835	3190	311
Patients affected by AEs	1164 (37.2%)	902 (48%)	251 (49.4%)
Most common toxicity	Infection (17.5%)	Infective episodes (32.3%)	Hepatic toxicity (40.2%)
Second most common toxicity	Methotrexate encephalopathy (8%)	Hepatotoxicity (28.2%)	Gastrointestinal toxicity (12.4%)
Third most common toxicity	Septicaemia (5.8%)	Gastrointestinal toxicities (20.4%)	Neurological toxicity (8.7%)
Non-relapse toxic mortality	35 (1.1%)	69 (3.7%)	N/A

The table shows the top three most common toxicities and alongside mortality rates because of treatment (Refs 23, 24, 25).

Chemoprotection induced by the leukaemia microenvironment is important in conferring treatment protection to cancer cells via mechanisms that include leukaemia cell–BM niche interactions and malignant dormancy (Refs 26, 27). ALL chemotherapies include DNA damaging and spindle poisons, which target the S and M phases of the cell cycle. These therapies rely on targeting actively cycling leukaemia cells, and therefore are ineffective against dormant cells which consequently lead to treatment resistance and relapse (Refs 28, 29). To improve efficacy of treatment and limit treatment failure and relapse, approaches including targeted therapy, immunotherapy and gene therapy are being explored.

Targeted therapy includes risk stratification, an approach where patients are grouped based on disease risk or therapy response from diagnostic tests. In a clinical trial for paediatric ALL (JPLSG MLL-10 trial), patients were stratified into three risk groups according to their *KMT2A* gene rearrangement status (KMT2A-r), age and presence of central nervous system (CNS) leukaemia (Ref. 30). High-dose cytarabine was given to KMT2A-r patients with haematopoietic stem cell transplant (HSCT) option being reserved for high-risk patients. Consequently, this removed the requirement for HSCT in patients with KMT2A-r (Ref. 30). Although patient stratification has contributed to the improved survival rates for paediatric ALL, intensifying chemotherapy attains a plateau where there is no additional benefit to patients but only an increased toxicity exposure. To overcome the limitations of targeted therapy, novel approaches need to be incorporated into the treatment protocol.

Immunotherapies have been explored to overcome the challenges presented by conventional targeted therapies. For example, blinatumomab presented promising results in a phase I/II trial with paediatric patients with relapsed/refractory ALL (Ref. 29). In a phase III trial in paediatric patients with B-ALL at high risk of relapse, blinatumomab was superior to conventional consolidation therapy (Ref. 31). However, blinatumomab presents unique and significant toxicities of neurological events and cytokine release syndrome (CRS), which includes pyrexia, headache, nausea, fatigue and hypotension, although these findings were presented from adults with relapsed B-ALL (Ref. 32). CRS has been seen to be infrequent in low minimal residual disease (MRD) settings and most neurological events could be reversed through interrupting infusions (Ref. 33), suggesting that blinatumomab could be effective with minimal toxicity in patients with low MRD, although alternatives would be needed in other patients.

Gene therapy is another emerging route to overcome the challenges of conventional therapies. T-cell therapy involves genetically engineering chimeric antigen receptor (CAR) T cells, coupling an anti-CD19 domain to intracellular T-cell signalling domains of the T-cell receptor, which redirects cytotoxic T lymphocytes to cells expressing the CD19 antigen, in B cell leukaemia (Ref. 34). Anti-CD19 CAR T-cell therapy, tisagenlecleucel, has been FDA-approved after high remission rates were found in patients with ALL and while severe toxicities were observed these effects were reversible (Refs 35, 36).

## The roles of cadherins in the leukaemia microenvironment

Classical cadherins are a calcium-dependent adhesion molecule family, grouped into type-I and type-II subgroups based on the molecular features of their interactions via the cadherin motifs (Ref. 37). Neural (N)-cadherin (CDH2) and epithelial (E)-cadherin (CDH1) are type-I cadherins which are characterised by the cell adhesion recognition motif His-Ala-Val (HAV) in their first extracellular domain (Refs 38, 39). CDH1 is a tumour suppressor protein which plays an important role in regulating tissue homoeostasis by modulating permeability barriers (i.e. tight junctions) between compartments, and the functional state of CDH1 determines metastatic potential (Ref. 40). Functional activity of CDH1 can be modified in response to environmental factors and CDH1 can be activated by monoclonal antibodies to inhibit metastasis at multiple stages of the metastatic cascade (Ref. 40). CDH2 is typically known for its role in morphogenetic processes in health such as during the formation of cardiac and neural tissue, and in diseases such as solid tumours. Moreover, recent research indicates overexpression of *CDH2* in HSCs show increased HSC attachment to BM endosteal surfaces (Ref. 5). In disease, loss of *CDH1* and upregulation of *CDH2* in cancer cells leads to metastatic dissemination and activation of several epithelial–mesenchymal transition (EMT) transcription factors (Ref. 41). EMT is a cellular morphogenetic transition from a non-motile, epithelial phenotype into a migratory, mesenchymal-like phenotype and is thought to be a driving force in tumourigenesis and metastasis (Refs 42, 43, 44, 45, 46). CDH2 has been identified as an important molecule of interest in leukaemia. A recent study demonstrated that this adhesion molecule was upregulated in leukaemia cells primed by BM niche cells (Ref. 4). Furthermore, MILE study and Bloodspot database showed that multiple haematological malignancies exhibited *CDH2* upregulation compared with healthy BMs (Refs 47, 48).

On a related note, osteoblast (OB)–cadherin (CDH11) a type-II cadherin (Ref. 35) important in the formation of the neural crest (NC) cells, has been further shown in disease models to cause tumour growth, cell survival and EMT (Refs 49, 50, 51). It has been further suggested that intracellular downstream signalling of CDH11 is essential for maintenance and survival of premigratory NC cells. In addition, cells require CDH11 for physiological cell–cell, adhesion-related EMT in the preparatory steps prior to migration (Ref. 52). However, the biological role of CDH11 in leukaemia has not yet been explored.

## The role of CDH2 in the BM niches and in chemoprotection

Biological systems are complex where their complexity is characterised by multicellularity, degeneracy and redundancy of the component cell types. The BM is a viscous tissue within the bone comprised of two well-defined niches – endosteal and perivascular, where HSCs are found in close proximity to OB and endothelial cells (ECs) (Ref. 53). All blood lineages and immune cells are derived from the common precursor, HSCs (Ref. 54), which retains the ability for both multipotency and self-renewal (Ref. 55). The two niches are intertwined to create a functional microenvironment, that facilitates cell communication during HSC development consequently helping to maintain the full blood cell forming potential of HSCs (Ref. 55).

It has been well-established that the endosteal niche is filled with mesenchymal stromal cells (MSCs), osteoprogenitor cells, pre-OBs, mature OBs, osteocytes and osteoclasts (Ref. 56). OBs play an important role in maintaining a functional microenvironment and are involved in stem cell quiescence and proliferation (Ref. 57). For example, SDF-1*α* in OBs is associated with HSC mobility (Ref. 58). In disorders that affect HSCs, such as myelodysplastic syndrome, it has been shown that suppressing osteogenic differentiation from MSCs leads to their impairment in supporting HSCs (Ref. 59). Non-collagenous bone matrix proteins, such as osteopontin and osteocalcin, regulate cell migration and bone mineralisation and are believed to be linked to cell proliferation, osteogenic differentiation and angiogenesis; however, these processes are yet to be defined in leukaemia cell biology (Ref. 60).

Because of inaccessibility of reliable animal models, the niche microenvironment of ALL has not been well-established. However, the remodelling of the BM vasculature following AML leukaemogenesis has been studied and it was found that AML cells aid the niche transformation into a preferential leukaemia microenvironment. These changes are anatomically diverse; although vasculature in the endosteum was lost through disease progression, central vessels survived with compromised function. This process was thought to be because of the production of pro-inflammatory and anti-angiogenic cytokines from AML cells in the endosteal lining which degrade the surrounding endothelium, as well as stromal osteoblastic cells, together leading to the reduced capacity to support HSCs. Vasculature was maintained in T-ALL murine models suggesting this vascular remodelling is specific to AML (Refs 26, 61). The inflammatory cytokine, TNF-*α*, secreted by AML cells, directly induces E-selectin which plays a role in promoting malignant cell survival, proliferation and chemoresistance (Ref. 27). AML engraftment also induces exogenous nitric oxide overproduction, which affects HSC motility and increases HSC activation leading to reduction in their repopulating activity (Ref. 26). Increased vascular leakiness was observed in AML xenografts after induction therapy, leading to poor drug delivery and the formation of areas with low perfusion rates, where leukaemia cell migration resulted in microenvironment-induced treatment resistance (Ref. 26).

Peri-arteriolar stromal cells which are innervated by the sympathetic nervous system and express neural markers NG2 and nestin (NG2^+^/nestin^+^ MSCs), have previously been found to control HSC quiescence and haematopoiesis (Ref. 62). The BM is known to be the site of dormant-disseminated tumour cells (DTCs) and Nobre *et al*. (Ref. 63) found that NG2^+^/nestin^+^ MSCs drive DTC dormancy which indicate that the perivascular niche is important for both HSC and DTC dormancy. NG2^+^/nestin^+^ MSCs produce TGF-*β*2 and BMP7, which signal a quiescent pathway through TGFBRIII and BMPRII, thereby activating SMAD, p38 and p27 pathways leading to dormancy (Ref. 63). Treatment induced damage of endosteal and perivascular niches have also been reported (Ref. 64). Further research determined that leukaemia cells have an important function in the development of a new therapy-induced niche formation. Following treatment, secretion of cytokines and growth factors were found to increase in the microenvironment likely because of secretion by the leukaemia cells (Ref. 64). Indeed the leukaemia niche has been reported to be transient, beginning initially as nestin^+^ cells maturing into *α*-SMA^+^ cells before terminating with fibre residues (Ref. 64).

**Figure 1.**
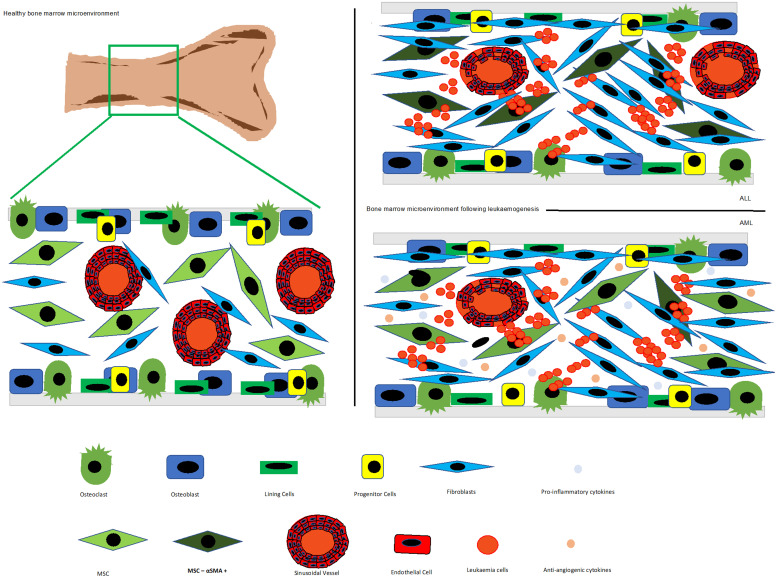
Schematic diagram of the BM microenvironment under normal conditions and following leukaemogenesis and treatment in AML (top right) and ALL (top left). After leukaemogenesis and treatment, the microenvironment is remodelled, pro-inflammatory and anti-angiogenic cytokines are produced resulting in the loss of vasculature in the endosteal and osteoblastic cells. Adapted from Refs 26, 27, 53, 54, 55, 56, 57, 58, 59, 60, 61, 62, 63.

In keeping with these studies, recent research shows upregulation of CDH2, a known marker of EMT, in niche-primed leukaemia cells. This study demonstrated that knockdown of CDH2 in leukaemia cells reduce their proliferation while increasing sensitivity to dexamethasone treatment (Refs 3, 4). Under physiological conditions, CDH2 plays a role in osteogenesis in the endosteal niche, specifically in maintaining the precursor OB pool (Ref. 65). CDH2-mediated interactions with OBs are thought to play a role in supporting HSC function, with HSC–OB cell interactions enabling adhesion of HSCs to cells present in the endosteal niche (Refs 5, 66). CDH2 is also expressed by various cell types associated with the HSC niche (Fig. 1), including stromal cells in the endosteal niche, and ECs and their associated pericytes in the microvascular of the perivascular niche (Ref. 5).

*CDH2* upregulation has been reported in human leukaemic BMs (Ref. 4). A recent study has shown that *CDH2* upregulation by niche-primed leukaemia is associated with increased cancer proliferation and acquisition of treatment resistance and importantly this interaction is druggable using the CDH2 antagonist ADH-1 (Refs 3, 4). In adult AML CDH2 supports tumour growth and aids in maintaining self-renewal characteristics of leukaemia stem cells (LSCs), as CDH2^+^ cells have been found to engraft on NOD/SCID mice at a higher proportion than CDH2^−^ cells (Ref. 6). CDH2 is also thought to support microenvironment-induced treatment protection in AML (Ref. 67). Indeed, adhesion interactions between LSCs and the BM microenvironment activate signalling cascades, which regulate functions including cell survival, evasion of apoptosis and cell dormancy. LSC interactions with the BM microenvironment enable them to evade the cytotoxic effects of chemotherapeutic agents, suggesting there is a reliance on adhesive interactions between AML LSCs and the BM for chemoprotection (Refs 5, 68). *CDH2* overexpression in HSCs decreases in vitro cell division rate, this is likely because of the sequestration of the CDH2 binding, intracellular *β*-catenin to the plasma membrane, thus suppressing its activity as a transcription factor in the nucleus (Refs 6, 38). In support of this, adult AML BM contains CDH2^+^ LSCs which are found in a quiescent state in G0/G1 cell cycle arrest, which renders them less sensitive to chemotherapy (Refs 6, 69). Lastly, in adult AML, CDH2 is also thought to play a role in drug resistance, CDH2^+^ LSCs were found to have a higher IC_50_ of VP-16, an anti-leukaemia therapeutic drug, than the CDH2^−^ population (Refs 6, 70).

## Pathways associated with CDH2

**Figure 2.**
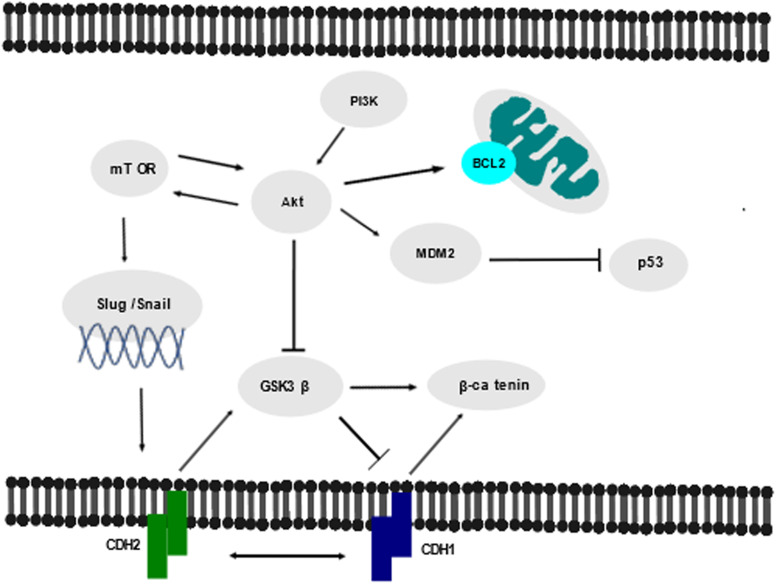
Schematic diagram of the pathways and transcription factors associated with CDH2, including the PI3K/Akt/mTOR pathway and the Wnt/*β*-catenin pathway. Arrows represent activation; bars represent inhibition, double-ended arrows in the pathway indicate upregulation of a molecule results in downregulation of the other and vice versa. Adapted from Refs 71, 72, 73, 74, 85.

There are many pathways that are associated with CDH2 in various malignancies. Two pathways of relevance to this review are the Wnt/*β*-catenin pathway and the phosphoinositide 3-kinase (PI3K)/Akt/mammalian target of rapamycin (mTOR) pathway, as detailed in Figure 2. It is of note, that there is limited research into these signalling pathways in acute leukaemia, clearly indicating an area warranting further exploration.

Hyperactivation of the PI3K/Akt/mTOR signalling pathway has been reported in 88% of ALL patients and is associated with poor prognosis and chemotherapeutic resistance (Ref. 74). The PI3K–Akt–mTOR pathway is important for haematopoietic cells, regulating functions such as HSC proliferation, differentiation and survival, and is furthermore constitutively activated in AML cells (Refs 78, 79). The presence of PI3K/Akt/mTOR pathway has been well-established in solid tumours and dimerisation and phosphorylation of PI3K leads to the downstream activation of Akt. Akt stimulates cell survival by upregulating mouse double minute 2 homologue (*MDM2*), which inhibits *p53*, and upregulates *BCL2*, both leading to inhibition of apoptosis. Akt activation subsequently triggers the phosphorylation of mTOR (Ref. 81). mTOR is a conserved serine/threonine kinase that belongs to the PI3K-related kinase family and has also been well-established in solid tumours. It is a constituent of two signalling complexes, mTORC1 involved in mRNA translation and protein synthesis and mTORC2 which controls cell survival and migration (Refs 82, 84, 85). There is evidence to link p70S6K to the Akt/mTOR pathway in AML (Ref. 77). In solid tumours p70S6K activates the transcription factors, slug and snail, which downregulates CDH1 and upregulates CDH2, leading to EMT (Ref. 75).

Dysregulation in the Wnt/*β*-catenin pathway can lead to initiation and progression of cancer, including haematological malignancies, and *β*-catenin activation has been found to contribute to ALL and AML drug resistance (Refs 71, 72, 73, 83). The inactivation of *GSK3β* from Akt-dependent phosphorylation prevents *β*-catenin phosphorylation, leading to the activation of *β*-catenin-independent genes and uncontrolled cell proliferation (Ref. 71). CDH2 regulates Wnt/*β*-catenin signalling, a conserved pathway that plays a role in physiological processes, including differentiation, proliferation and cell fate determination. CDH1 is known to inhibit the activation of the Wnt pathway, and in ALL CDH1 has been shown to be decreased, indicating Wnt pathway activation (Ref. 76). Additionally in ALL, Akt has been shown to inhibit GSK3*β* leading to the activation of *β*-catenin (Ref. 80).

## Therapeutic approaches targeting CDH2

### Exherin (ADH-1)

**Figure 3.**
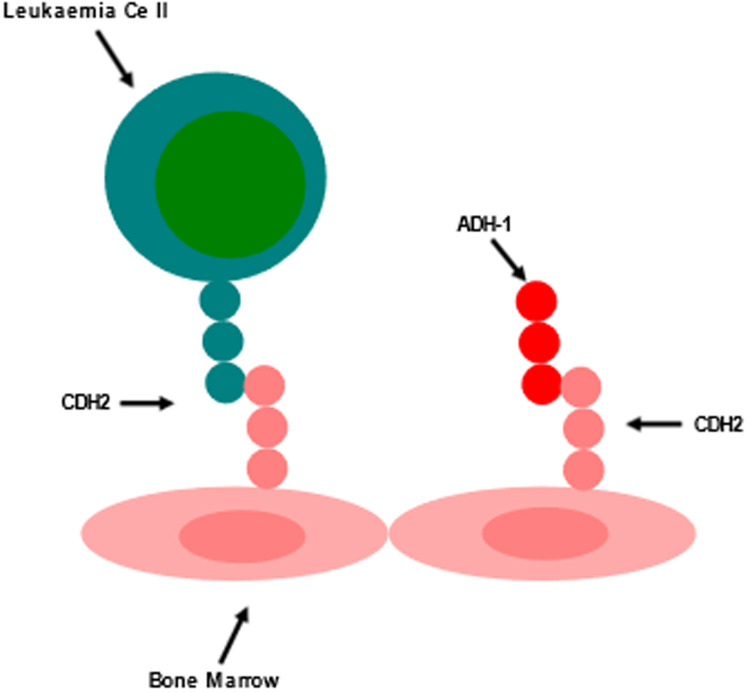
ADH-1 competitively binds to CDH2 on BM cells, preventing leukaemia–niche cell binding of leukaemia cells within the BM microenvironment. Adapted from Refs 38, 86.

The CDH2 antagonist ADH-1 is a cyclic pentapeptide which competitively inhibits CDH2 (Fig. 3), as it contains the cadherin cell adhesion recognition sequence HAV (Refs 38, 86). The proposed mechanism of action of ADH-1 in cancer is that it results in apoptosis in vitro, and causes inhibition of tumour cell migration in addition to altering the tumour vasculature in vivo (Refs 87, 88, 89). Pal *et al*. (Ref. 4) found that ADH-1 showed high efficacy in vitro and in vivo against patient-derived ALL cells, where ADH-1 reduced proliferation of ALL cells in vitro, as indicated by a reduced number of blasts in the S phase of the cell cycle (Ref. 4). This research further assessed ADH-1 activity on CDH2 knockdown ALL cells, where ADH-1 treatment sensitivity was confirmed only in the wild-type ALL cells that did not harbour the CDH2 knockdown, thereby corroborating specificity of ADH-1 against CDH2 and suggesting against the likelihood of off-target effects (Ref. 4). This study further validated ADH-1 to show efficacy both as a single agent and in combination with dexamethasone, in a patient-derived xenograft (PDX) mouse model, where addition of ADH-1 to dexamethasone did not result in any additional toxicity (Ref. 4). Of note, ADH-1 is an FDA-approved compound with ‘orphan drug’ status for use in melanomas (Ref. 90), and ADH-1 treatment in patients with solid tumours was well tolerated resulting only in a few adverse events, most of which were grade 1 or 2, thereby showing a better tolerance than most current treatments (Ref. 91). These findings indicate ADH-1 to be a potentially promising therapeutic agent that could be repurposed from solid cancers to leukaemia treatment.

In addition, ADH-1-modified liposomes (A-LP) have been successfully constructed with the aim of enhancing chemotherapy efficacy and preventing metastasis and was tested using a PTX-resistant breast cancer cell line, MCF7 PTX-R, which was established into a tumour model using subcutaneous inoculation into the right flanks of female BALB/c nude mice. Results found that cellular uptake was increased because of the CDH2 expressed after EMT in the MCF7 PTX-R cells (Ref. 91). Treatment with the A-LP showed cancer cells to have an increased chemo-sensitivity, with EMT to be somewhat suppressed.

ADH-1 has been further shown to improve immunotherapy by tumour-infiltrating lymphocyte (TIL)-related treatment. The immune dysfunction mechanism including programmed death ligand-1 (PDL-1) and indole amine 2,3-dioxygenase (IDO-1) induces apoptosis, both PDL-1 and IDO-1 are increased after EMT and immunosuppression is enforced. Therefore targeting CDH2 improved the efficacy of TIL-related treatment by decreasing PDL-1 and IDO-1, and indeed ADH-1 with TIL-treatment reduced tumour size and increased survival in the mouse models (Ref. 92). Although ADH-1 has been documented in cancer pre-clinical studies and solid tumour clinical trials, in-depth mechanism of action of this drug remains unexplored. Further research needs to be conducted to develop an in-depth understanding of ADH-1, including scrutiny of any possible mechanisms of resistance that could arise following ADH-1 treatment. In addition, several next-generation antagonists, including small-molecule inhibitors of CDH2 are being developed (Ref. 38) and their role as potential anti-leukaemia treatment needs to be investigated.

### CDH2 small-molecule antagonists

Much less is known concerning the biological effects of other types of CDH2 antagonists, as they have not been extensively developed for use as cancer therapeutics (Ref. 38). A large number of non-peptidyl peptidomimetics of ADH-1 have been recently identified (Refs 93, 94), for example, the small-molecule LCRF-0006 is an ADH-1 peptidomimetic that inhibits CDH2 function, induces apoptosis in multiple myeloma (MM) and synergises with bortezomib to enhance MM cell death in vitro (Ref. 93).

Non-peptide peptidomimetics of the CDH2 Trp-containing amino-terminus have also been discovered and are being developed as cancer therapeutics (Refs 38, 95). In particular, the peptidomimetic-designated Compound 15, a piperidin-4-amine which acts as a CDH2 antagonist, has been shown to induce apoptosis of MM, glioblastoma and pancreatic cancer cells, as well as fibroblast and cancer-associated death in vitro (Refs 38, 96). However, the ability of this small molecule to affect leukaemia blast viability as well as its mechanism of action remains unexplored.

## Targeting other pathways in combination with CDH2

Although targeted therapy underpinning oncogene addiction has shown great promise in cancer treatment, it is associated with emergence of treatment-resistant clones. Combinatorial therapies target multiple cancer pathways, and thereby aim to mitigate occurrence of treatment resistance. Furthermore, up to 40% of ALL patients present with CNS involvement, because of the ability of leukaemia cells to penetrate the blood–brain–barrier (BBB) (Ref. 97). Although it is now well-established that achieving CNS clearance in ALL is essential for long-term disease cure, CNS-directed therapy is associated with significant toxicity (Ref. 98). This highlights need for new and improved combinatorial treatments in ALL to prevent treatment resistance and mitigate treatment toxicity. Indeed combination therapies containing dexamethasone, a glucocorticoid routinely used to treat ALL, with venetoclax or ADH-1 have been shown to increase leukaemia-free long-term survival in pre-clinical mouse models and patients (Refs 4, 99, 100). Furthermore, ADH-1 and dexamethasone have been found to show high efficacy when tested in combination on PDX mouse models transplanted with high risk ALL. The ADH-1/dexamethasone combination was found to significantly reduce the proportion of leukaemia blasts in vivo compared with the dexamethasone-only arm, and moreover addition of ADH-1 to dexamethasone did not result in any additional toxicity (Ref. 4).

Adults with BCR-ABL^+^ ALL have poor prognosis; therefore, dexamethasone was tested in a triple combination with venetoclax and tyrosine kinase inhibitors (TKIs), imatinib or dasatinib. Both combinations were shown to be superior to single agents and double combinations in terms of tumour size and survival, although the combination with dasatinib was shown to be more effective (Ref. 100). Researching the value of adding ADH-1 to a dexamethasone/venetoclax/TKI is warranted especially in high risk and/or refractory disease to assess if this combination would improve efficacy and minimise emergence of treatment resistant clones (Ref. 100). Other drugs and pathways where adding ADH-1 as a combinatorial treatment might be valuable is as discussed below.

Dysregulation in the PI3K/Akt/mTOR pathway has been well-established as a component of AML pathogenesis. Many pharmacological inhibitors within this pathway have been evaluated in preclinical settings; however, there is yet to be meaningful clinical effectiveness of inhibition of this pathway for AML. Buparlisib is an oral pan-class I PI3K inhibitor, and has completed a phase I trial of patients with acute leukaemia and at doses of 80 mg/day was found to be tolerable with a modest single-agent efficacy. Buparlisib has also been seen to cross the BBB which is of importance in ALL with CNS infiltration (Refs 101, 102).

Idelalisib is a PI3K-*δ* inhibitor, more specifically p110*δ* a primary PI3K isoform in B cells and has shown activity in lymphoid malignancies and been FDA-approved for relapsed chronic lymphocytic leukaemia (CLL), follicular lymphoma and small lymphocytic lymphoma (Ref. 103). Haematological malignancies such as relapsed CLL, follicular lymphoma and small lymphocytic lymphoma have been observed to depend on pre-B cell receptor signalling, which can also be seen in the majority of TCF3-PBX1 BCP-ALLs. The specificity of idelalisib to p110*δ*, results in a low toxicity profile, making it a promising therapeutic for TCF3-PBX1 BCP-ALL patients (Refs 104, 105). Interestingly, significant CDH2 upregulation in TCF3-PBX1 leukaemic BM combined with high ADH-1 efficacy seen in TCF3-HLF PDX samples would suggest that combining a CDH2 antagonist with idelalisib might be potentially beneficial.

mTOR inhibitors have shown promise in preclinical models of ALL through direct inhibition of tumour cell growth and reversal of glucocorticoid resistance and have demonstrated in vitro synergy with dexamethasone (Ref. 106). Everolimus presents these preclinical characteristics as a single agent, making it a good candidate for combination treatment. Moreover, there is a phase II study of everolimus in combination with vincristine, prednisone, pegaspargase and doxorubicin in relapsed ALL (Ref. 107). Everolimus was also tested in chronic myeloid leukaemia patients and found that in combination with imatinib, treatment was effective in both sensitive and resistant cases (Ref. 108).

BEZ-235 is a dual pan-class I PI3K and mTOR inhibitor that has been tested in adult patients with relapsed/refractory acute leukaemia. Clinical development of BEZ-235 has been terminated because of suboptimal pharmacokinetic properties. Although this study found that efficacy observed in ALL patients warrant further clinical exploration into dual PI3K/mTOR inhibitors, in particular patients with Ph + BCP-ALL or T-ALL may benefit from these treatments (Ref. 109). Given the link between CDH2, mTOR and EMT all of which play an important role in cancer biology (the role of EMT in non-epithelial cancers such as leukaemia is an emerging concept (Ref. 110)), including niche-driven leukaemia cell behaviour, combining a CDH2 antagonist with mTOR inhibitors may have a potential therapeutic benefit.

Despite the role Wnt plays within acute leukaemia and its connection with CDH2, there has not been any clinical or preclinical testing with Wnt inhibitors, suggesting a potential area of further research, some pre-clinical antagonists are highlighted in Table 2 (Refs 111, 112, 113, 114, 115). Table 2 highlights inhibitors that have been tested against other cell lines and malignancies.

**Table 2.** List of the therapeutics, their targets and their progressions through clinical trials

Name	Target	Clinical trial progression?
ADH-1	CDH2	Phase II (solid tumours)
AZD2014	mTORC1 and mTORC2	Phase II (solid tumours)
AZD8055	mTORC1 and mTORC2	Phase I (halted development)
BEZ-235	PI3K and mTOR	Phase I (ALL), phase II (solid tumours)
Buparlisib	PI3K	Phase I
Capmatinib	Wnt	Pre-clinical
Compound 15	CDH2	Pre-clinical (solid and haematological cancers)
Dasatinib	Tyrosine kinase	FDA-approved for paediatric chronic myeloid leukaemia
Dexamethasone	Glucocorticoid receptors	FDA-approved
Everolimus	mTOR	FDA-approved (solid tumours)
Idelalisib	PI3K-*δ*	FDA-approved for chronic lymphocytic leukaemia
Imatinib	Tyrosine kinase	FDA-approved for chronic myeloid leukaemia
Isoquercitrin	Wnt	Pre-clinical
IWP-4	Wnt	Pre-clinical
XAV-939	Wnt	Pre-clinical
Venetoclax (ABT-199)	BCL-2	FDA-approved for AML

## Conclusion

In conclusion, CDH2 is an important molecule in both the healthy and malignant BM microenvironment, supporting both non-malignant haematopoietic cells and leukaemia cells. CDH2 supports tumour growth and promotes microenvironment-mediated treatment protection, decrease cell division rate and potentially plays a role in cancer dormancy. ADH-1, a first generation CDH2 inhibitor used in solid tumour clinical trials, demonstrated a well-tolerated toxicity profile and therefore may be an ideal candidate for combinatorial treatment in acute leukaemia. It is important to note that only CDH2 antagonists target the extracellular domain of cell surface receptors making them a unique class of therapeutic drugs. Furthermore, targeting other pathways that are associated with CDH2 may overcome environment-mediated drug resistance and may help reduce the rate of relapse in paediatric acute leukaemia. Next-generation CDH2 antagonists such as small-molecule inhibitors with improved potency and formulation are emerging as a unique class of anti-cancer therapeutics. These are potentially capable of targeting microenvironment-mediated malignant dormancy and treatment resistance in leukaemia and following in-depth preclinical and clinical validation may provide improved and low toxicity treatment options in paediatric leukaemia.
